# Can Gossip Buffer the Effect of Job Insecurity on Workplace Friendships?

**DOI:** 10.3390/ijerph16071285

**Published:** 2019-04-10

**Authors:** Lixin Jiang, Xiaohong Xu, Xiaowen Hu

**Affiliations:** 1 School of Psychology, University of Auckland, 23 Symonds Street, CBD, Auckland 1010, New Zealand; 2 Department of Psychology, Old Dominion University; 250 Mills Godwin Life Sciences Bldg, Norfolk, VA 23529, USA; x3xu@odu.edu; 3 Business School, Queensland University of Technology, 2 George Street, Brisbane, QLD 4000, Australia; xiaowen.hu@qut.edu.au

**Keywords:** job insecurity, workplace friendships, gossip, stress coping

## Abstract

Although previous research has documented a host of negative consequences of job insecurity, workplace interpersonal relationships have rarely been considered. This omission might be caused by the application of broad stress theories to the job insecurity literature without taking a nuanced perspective to understand the nature of job insecurity. To address this issue, we conceptualized job insecurity as a threat to employee social acceptance by their employer. This conceptualization, therefore, allows us to apply the multimotive model of social rejection to investigate a previously-overlooked outcome of job insecurity—workplace friendships. Specifically, we investigated the relationship between both job feature insecurity and job loss insecurity with workplace friendships. Based on stress coping theory and the fundamental differences between job feature insecurity and job loss insecurity, we further proposed that employees’ tendency to engage in positive gossip buffers the negative impact of job feature insecurity on workplace friendships, whereas employees’ tendency to engage in negative gossip buffers the negative impact of job loss insecurity on workplace friendships. Data collected from 286 working adults from Mturk supported our hypotheses. Our study opens the door for future research to take a more nuanced approach when examining nontraditional consequences of job insecurity.

## 1. Introduction

As employers are building an agile workforce, gone are the days of lifetime-guaranteed employment. Workers at every level are experiencing increased uncertainty and are unsure how much longer they will be employed [1]. Indeed, recent years have observed an increase in nonstandard work arrangements (e.g., employees of temporary help agencies and contract companies, independent contractors, and on-call workers [2]). For example, surveys indicate that 18.7% of adults in the U.S. work in nonstandard arrangements [3], and the percentage of nonstandard arrangement grew to 15.8% in 2015 [4]. Consequently, job insecurity is called “the disease of the 21st century” [5] and “the single largest cause of uncertainty in the high-risk society” [6] (p. 160).

Living in chronic fear of that their job can undergo significant changes at any time (i.e., job insecurity) can be devastating, putting job-insecure workers at risk for mental [7] and physical illness, anxiety, and depression [8,9,10]. The “long arm” impact of job insecurity extends beyond employees to their family members, including their spouse [11] and children [12,13]. Indeed, a recent meta-analysis revealed over 40 adverse outcomes of job insecurity [8].

However, what has been missing from previous meta-analyses and empirical studies is a focus on interpersonal consequences of job insecurity [14]. Such an omission is not an accident; rather, it is primarily caused by the simple conceptualization of job insecurity as a work stressor without deeply considering the nature of job insecurity. Indeed, job insecurity is most commonly conceptualized as a work stressor that can be understood via (a) latent and manifest benefits of work [15], (b) cognitive appraisal theory [16], (c) conservation of resources theory [17], and (d) the job demands–control model [18]. Although these broad theories enable us to understand a wide range of work stressors and their negative consequences, they tend to adopt a “one-size-fits-all” approach, ignoring the nuanced differences between each work stressor. Consequently, merely categorizing job insecurity as a widespread work stressor [1] limits our understanding of job insecurity and misses out the opportunity to shed light on the essence of job insecurity that is different from other commonly encountered work stressors (e.g., work overload). Hence, we took a different perspective by conceptualizing job insecurity as a threat to employee social acceptance by their employer. This conceptualization, therefore, allows us to apply the multimotive model of social rejection [19] to investigate some previously-overlooked outcomes of job insecurity (e.g., interpersonal relationships).

Specifically, drawing upon the multimotive model of social rejection [19], we examined an interpersonal outcome of job insecurity—workplace friendships. Although workplace subordinate–supervisor relationships have been extensively examined, scholars rarely pay attention to coworker relationships and friendships. This lacuna is surprising, because most people spend a significant portion of their life at work, and a more significant number of working hours is spent with employees’ coworkers and colleagues than their supervisor. Thus, workplace friendships should have far-reaching implications to employees and their organization. Indeed, workplace friendships exert their influence on factors within [20,21,22] and beyond the organization sphere [23]. As such, establishing the relationship between job insecurity and workplace friendships also contributes to our understanding of the development of workplace friendships.

Because the vast majority of empirical research on job insecurity has taken a global perspective and defined job insecurity as the threat to the continuity of one’s employment [1], Greenhalgh and Rosenblatt [24] posit that the “loss of valued job features is an important but often overlooked aspect of job insecurity” (p. 441). To address this issue, Hellgren, Sverke, and Isaksson [25] proposed the distinction between qualitative job insecurity (i.e., job feature insecurity; the threat to the continuity of important job features) and quantitative job insecurity (i.e., job loss insecurity). Since then, more research has been done about job feature insecurity. Nevertheless, compared to investigations on job loss insecurity, there are significantly fewer studies on job feature insecurity [1,26], with even less research comparing these two forms of job insecurity. However, it is important to examine both types of job insecurity because the transformation of work and technological advancement might make job feature insecurity more prevalent [1]. Moreover, because of their key difference in the continuity or the loss of organizational membership [14], comparing and contrasting these two types of job insecurity is necessary. Due to the multifaceted reality of job insecurity [1,24], we examined the relationships of both job feature insecurity and job loss insecurity with workplace friendships.

Integrating the multimotive model of social rejection [19] and stress coping theory [27], we propose that the tendency to engage in gossip in the workplace—an emotion-focused coping style—might buffer against the negative impact of job insecurity on workplace friendships. Moreover, we consider the key difference between job loss insecurity and job feature insecurity and detail the potential functions of engaging in different types of workplace (i.e., positive vs. negative) gossip in the times of job insecurity. We argue that the impact of different forms of job insecurity—job feature insecurity and job loss insecurity—are subject to one’s tendency to engage in different types of gossip—positive and negative gossip. Specifically, job feature insecurity allows individuals to remain in the same organization; the negative impact of job feature insecurity might be attenuated by employees’ tendency to engage in positive gossip, which allows them to remain on good terms with their colleagues. Thus, individuals who tend to engage in positive gossip at work might be better at coping with job feature insecurity. By contrast, job loss insecurity implies that employees might lose their organizational membership; individuals who are higher in their tendency to engage in negative gossip might be able to effectively deal with the negative impact of job loss security by damaging the reputation of their potential competitors in the workplace. As such, we propose the buffering effect of the tendency to positive gossip on the relationship between job feature insecurity and workplace friendships and the buffering effect of the tendency to negative gossip on the relationship between job loss insecurity and workplace friendships. To conduct a preliminary examination of our proposals, we recruited a convenience employee sample from Amazon’s Mechanical Turk.

Together, we advanced previous research in several ways. First, by conceptualizing job insecurity from the lens of the multimotive model of social rejection [19] and examining the negative implications of job insecurity with regard to workplace friendships, we expanded the outcomes of job insecurity to an interpersonal factor that has previously been ignored (i.e., workplace friendships). Second, we compared and contrasted two types of job insecurity—job loss insecurity and job feature insecurity. In doing so, we deepened our understanding of different forms of job insecurity. Third, drawing on stress coping theory [27] and the fundamental difference between job loss insecurity and job feature insecurity, we explored the attenuating effect of one’s tendency to engage in positive and negative gossip on the relationship between two types of job insecurity and workplace friendships. As such, we provided a fine-grained perspective on how different traits [28] might function to buffer the negative impact of different forms of job insecurity, thereby opening the door for future research to take a more nuanced approach when examining the consequences of job insecurity.

### 1.1. Job Insecurity and Workplace Friendships

The potential loss of continuity in a job situation can range from “permanent loss of the job itself” to “loss of some subjectively important feature of the job” [24] (p. 440). Specifically, job loss insecurity (i.e., quantitative job insecurity) involves the threat to the continuity of the job itself, while job feature insecurity (i.e., qualitative job insecurity) involves threats to the continuity of important job features [1,9,25,29,30,31]. Given the increasing prevalence of, but insufficient attention to, job feature insecurity [1], we examined both job loss insecurity and job feature insecurity. Specifically, we explored the implications of both types of job insecurity with regard to workplace friendships.

Workplace friendships represent a unique type of workplace relationships because of its voluntary nature and personalistic focus [23]. Unlike compulsory workplace relationships such as supervisor–subordinate relationships, workplace friendships are voluntary. Although employees typically cannot choose whom to work with, they have a choice about which of their colleagues to befriend. Workplace friendships are also featured by their personalistic focus in that employees come to know and treat each other as an individual being rather than simply another jobholder [32].

Both individual and contextual factors shape workplace friendships. For example, employees with perceived similarity in attitudes, values, and interests are more likely to develop friendships [33]. Research also suggests that physical proximity facilitates friendship development [34], while the loss of proximity (e.g., moving away) can lead to the dissolution of friendships [35,36]. Moreover, working together on shared projects and tasks and spending time together outside the workplace (e.g., having drinks after work) contribute to the development of workplace friendships [32].

The relationship between job insecurity and workplace friendships, however, has not been examined. We conceptualize job insecurity, the threat to the desired continuity and stability of employment [14], as a job situation where employees experience the threat to valued social acceptance by their employer [19]. Specifically, job loss insecurity represents the likelihood of social rejection by their current employer who might soon deny their organizational membership, while job feature insecurity signals social devaluation and lowered acceptance by their employer [19]. As such, both forms of job insecurity represent looming social rejection.

The multimotive model of social rejection [19] suggests that people likely experience three sets of motives after social rejection. The first set of motives emphasizes an increased desire for social connections. The second set of motives highlights the aggressive urges of people who are rejected to defend themselves or hurt those who have rejected them. The third set of motives posits that people who are rejected are also driven to avoid further rejection and its accompanying hurt. Consequently, they may withdraw from social contact from both the source of the rejection and others whose acceptance they doubt. Although people more or less experience these three motives simultaneously [19], we focus on the socially avoidant behavioral responses as consequences of job insecurity. Our reasoning is based on the findings that job insecurity is a chronic stressor [1], and the motive to withdrawal is most likely to occur when the rejection is perceived to be choric or pervasive [19]. Accordingly, employees who experience job insecurity as anticipated social rejection from their employer may avoid social contact in the workplace and report decreased friendships as a result.

Taking a purely pragmatic perspective, job-insecure employees who may expect to lose their employment in the near future (i.e., job loss insecurity) may see little value in interacting and socializing with those whom they may soon lose touch with. Continued interaction with someone whom one might lose contact with in the future might even be considered as waste of time and energy. As a result, withdrawal and avoidance is perhaps most likely when employees do not expect their relationships to last. Thus, withdrawal may be motivated by a desire to avoid additional investment in the current employer and their colleagues in the same organization. On the other hand, people may perceive themselves as worthless and inferior due to their perceived possibility of losing important job features (i.e., job feature insecurity [37]). Understandably, people who feel worthless or inferior are likely to believe that others will not value their friendships. As a result, they are motivated to avoid socialization with other people in the workplace. As such, we suggest a negative relationship between both types of job insecurity and workplace friendships. That is:

**Hypothesis** **1.** *Both job loss insecurity (H1a) and job feature insecurity (H1b) are negatively associated with workplace friendships*.

### 1.2. Buffering Effect of the Tendency to Gossip in the Workplace

The multimotive model of social rejection also proposes that individual differences (e.g., self-esteem) may influence how people predominantly respond to social rejection [19]. We expect the tendency to gossip in the workplace to be a dispositional moderator in the relationship between job insecurity and workplace friendships. Individuals often talk about others in the private as well as in the organizational settings. Indeed, people, across various ages and genders, spend approximately 65% of speaking time in everyday conversation on gossiping [38]. The content of such talk determines whether it is considered as positive or negative gossip. Specifically, when such talk about others is positive and complimentary, then positive gossip occurs. For example, a gossiper mentions a coworker’s excellent performance when the coworker is absent. By contrast, when such talk about others is negative and judgmental, then negative gossip occurs [39]. An example of negative gossip is that a gossiper discusses an absent coworker’s mistakes [40].

In the organizational setting, gossip has shown to have important functions [41,42,43]. For example, the tendency to gossip can be a way of coping when confronted with stressors [44]. Specifically, one’s tendency to gossip can be considered as *an emotion-focused coping style* because, unlike context-dependent coping behavior, one’s tendency to gossip is a trait-like individual difference [27,45,46,47,48,49,50,51,52] that may serve as a means of stress relief (i.e., emotion-focused coping) as opposed to addressing the source of the problem (i.e., problem-focused coping). Although emotion-focused coping strategies do not address the source of the problem [41,45], they are more effective when individuals have little control over the stressors [53,54,55,56,57,58]. Indeed, job insecurity represents such a low job-control situation as people do not know what can be done to adequately address the problem at hand [59]. For example, emotion-focused coping strategies were found to be more effective than a problem-focused coping approach in the wake of receiving a layoff notice [60]. Thus, the tendency to gossip, as an emotion-focused coping style, might attenuate the association between job insecurity and workplace friendships.

Moreover, considering the difference between job loss insecurity and job feature insecurity and the distinction between one’s tendency to positive gossip and negative gossip, we propose that the tendency to positive gossip may buffer the negative impact of job feature insecurity on workplace friendships, and the tendency to negative gossip might buffer the negative impact of job loss insecurity on workplace friendships [61]. One key difference between job feature insecurity and job loss insecurity lies in the continuity or the loss of organizational membership [24]. Given that job feature insecurity indicates the continuity of organizational membership, job-feature insecure employees retain the social status as employed people. By contrast, individuals with job loss insecurity suffer from the anticipation of becoming members of unemployed people [62]. Indeed, employees with job feature insecurity, compared to those with job loss insecurity, are still able to reap the latent and manifest benefits associated with employment, albeit to a lesser extent [15]. As such, job loss insecurity and job feature insecurity, which differ in terms of organizational membership, may dictate the effectiveness of positive or negative gossip that employees tend to engage in [28,63].

Specifically, we argue that tendency to positive gossip might be functional when employees experience job feature insecurity. Because job feature insecurity indicates the continuity of organizational membership, it is desirable for employees to display behaviors that align with the organization’s values and expectations [14], such as portraying themselves as “good soldiers” [64] and engaging in positive gossip. As such, tendency to positive gossip may allow individuals to effectively cope with job feature insecurity. By contrast, we expect that tendency to negative gossip might be functional when employees are threatened with the loss of organizational membership (i.e., job loss insecurity). To compete with potential rivals for limited positions in the organization, employees with job loss insecurity may decrease their helping behaviors directed at coworkers [65] and instead exhibit deviant behaviors towards coworkers (e.g., negative gossip) [14,66]. Indeed, given that job loss insecurity indicates the loss of organizational membership and employees may no longer see each other in the future, it might be desirable to engage in negative gossip by damaging the reputation of coworkers [66]. Therefore, one’s tendency to negative gossip may enable individuals to effectively deal with job loss insecurity. Together, both ways of coping (i.e., positive gossip or negative gossip) might give employees a sense of control in the stressful situation of job insecurity [67], and perceived control over a stressor can diminish the effect of stressors on strain [18].

**Hypothesis** **2a.** *The tendency to negative gossip moderates the relationship between job loss insecurity and workplace friendships in that the negative relationship between job loss insecurity and workplace friendships becomes weaker under high negative gossip compared to low negative gossip*.

**Hypothesis** **2b.** *The tendency to positive gossip moderates the relationship between job feature insecurity and workplace friendships in that the negative relationship between job feature insecurity and workplace friendships becomes weaker under high positive gossip than low positive gossip*.

## 2. Method

### 2.1. Participants and Procedures

In order to test our hypotheses, cross-sectional survey data of American employees were collected from Amazon’s Mechanical Turk, an online recruiting platform for human subject research. This study was approved as exempt by the first author’s Institutional Review Board (Review Category: Exempt-45 CFR 46. 1010 (b) (2); Protocol Number: 972680). In order to ensure data quality, we only recruited participants with a minimum of 95% prior approval rating across a minimum of 500 previously completed tasks [68]. We also implemented quality checks throughout the survey. Specifically, two attention check questions (e.g., “If you are reading this, please select ‘neither agree nor disagree’”) were scattered in the questionnaire.

We collected data from 300 employees. After excluding participants who failed the attention checks, the final dataset included 286 employee participants. Examination of the demographic characteristics of the sample revealed that the majority of participants were Caucasians (79%), and full-time (93%) and permanent employees (99%). About half of the participants were female (52%). On average, participants were 37 years old (*SD* = 10.62), worked for their current organization for six years (*SD* = 5.59), and spent 40 h at their work that was outside of Amazon’s Mechanical Turk (*SD* = 6.67).

### 2.2. Measures

#### 2.2.1. Job Loss Insecurity

The job security index (JSI) scale [69] was used to measure employee perceptions regarding the possibility of their job loss. Participants responded on a 3-point scale (*yes, don’t know, no*) measuring the extent to which 9 adjectives or phrases described the future of their job (e.g., “unpredictable”, “questionable”, “unknown”). Responses were scored such that higher numbers reflect higher levels of job loss insecurity. The Cronbach’s alpha reliability of the scale was 0.96.

#### 2.2.2. Job Feature Insecurity

We used five items from the job change insecurity dimension from O’Neill and Sevastos [70] to access job feature insecurity (e.g., “I am expecting unfavorable changes to my job”). The responses ranged from 1 (*strongly disagree*) to 7 (*strongly agree*). The Cronbach’s alpha reliability was 0.87.

#### 2.2.3. Tendency to Positive and Negative Gossip

We used four items [52] to assess one’s tendency to engage in negative gossip (α = 0.93; e.g., “At work, I talk with others about other people’s mistakes.”). We adapted the same measure to evaluate one’s tendency to engage in positive gossip (α = 0.93; e.g., “At work, I talk with others about other people’s achievement”). The responses ranged from 1 (*strongly disagree*) to 7 (*strongly agree*).

#### 2.2.4. Workplace Friendships

Three items adopted from Nielsen, Jex, and Adams [71] were used to evaluate employees’ workplace friendships. Specifically, employees indicated their levels of agreement with the following items: “I have formed strong friendships at work,” “I feel close to some of the people I work with,” and “I work with people I consider close friends” using the 7-point Likert scale ranging from 1 (*strongly disagree*) to 7 (*strongly agree*). The reliability of this scale was 0.95.

### 2.3. Control Variables

We controlled a number of demographic variables to examine whether the results of our model were still held after accounting for employees’ gender, age, full- vs. part-time employment, permanent vs. temporary employment, and the number of working hours per week, because previous research found that younger employees and permanent workers report lower levels of job insecurity [72], there is a gender difference in workplace friendships [73], and full-time workers might have more time to engage in workplace gossip than part-time workers.

## 3. Results

Table 1 presents descriptive statistics and zero-order correlations among the variables of interest. Both job feature insecurity and job loss insecurity were negatively related to workplace friendships.

Table 2 presents results of regression analyses. Both job loss insecurity (*β* = −0.28, *p* < 0.001) and job feature insecurity (*β* = −0.47, *p* < 0.001) was negatively related to workplace friendships, supporting Hypothesis 1.

**Figure 1:**
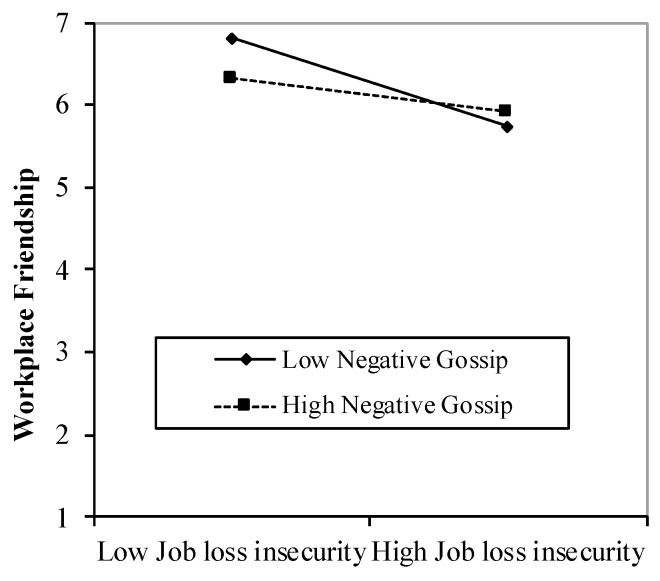
Buffering effect of the tendency to negative gossip on the negative relationship between job loss insecurity and workplace friendships.

Of primary interest to this study were the interaction results. As predicted by Hypothesis 2a, we found a marginal interaction effect between job loss insecurity and negative gossip on workplace friendships (*β* = 0.10, *p* = 0.062), which became significant after considering control variables (*β* = 0.12, *p* = 0.032; Step 3 in Table 2). The simple slope [74] of the regression of workplace friendships onto job loss insecurity under high negative gossip was only marginally significant (simple slope = −0.20, *t* = 1.78, *p* = 0.076). For those with low negative gossip, the relation between job insecurity and workplace friendships was significant (simple slope = −0.53, *t* = −4.83, *p* < 0.001). Thus, the finding that the relation between job loss insecurity and workplace friendships was stronger for those with low negative gossip than those with high negative gossip was consistent with our prediction (see Figure 1).

**Figure 2:**
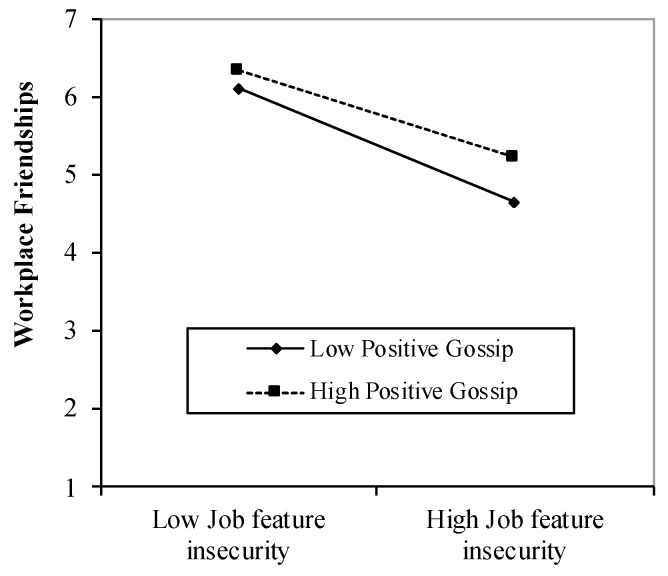
Buffering effect of the tendency to positive gossip on the negative relationship between job feature insecurity and workplace friendships.

On the other hand, we found a significant interaction effect between job feature insecurity and positive gossip on workplace friendships both with (*β* = 0.15, *p* = 0.018; Step 2 in Table 2) and without control variables (*β* = 0.13, *p* = 0.039; Step 3 in Table 2). Consistently, the simple slope analysis [74] in Figure 2 revealed that the association between job feature insecurity and workplace friendships was stronger for those with a low tendency to positive gossip (simple slope = −0.61, *t* = −8.56, *p* < 0.001) than those with a high tendency to positive gossip (simple slope = −0.42, *t* = −4.40, *p* < 0.001). Thus, we found support for Hypothesis 2b.

## 4. Discussion

Consistent with our theoretical predictions, we found that both job loss insecurity and job feature insecurity are negatively related to workplace friendships. Further, employees’ tendency to engage in positive gossip buffers against the negative impact of job feature insecurity on workplace friendships, whereas employees’ tendency to engage in negative gossip buffers against the negative impact of job loss insecurity on workplace friendships.

### 4.1. Theoretical Implications

Our study provides several theoretical implications. First, we contribute to the job insecurity literature by demonstrating that workplace friendship is another important outcome of job insecurity. In doing so, we respond to scholars’ call for investigating the negative consequences of job insecurity on employees’ interpersonal relationships in the workplace [14], which broadens our understanding of the implications of job insecurity. The findings that job loss insecurity and job feature insecurity are negatively related to workplace friendships suggest that simply conceptualizing job insecurity as yet another type of work stressors has limited our understanding of the true nature of job insecurity and that theoretical advancements in delineating the essence of job insecurity are needed.

Second, we found that the negative impact of job loss insecurity and job feature insecurity on workplace friendships are moderated by different dispositions—the tendency to engage in either positive or negative gossip. These findings suggest that ignoring the conceptual difference between job feature insecurity and job loss insecurity, or taking a global perspective to understand job insecurity, may lead to misleading conclusions regarding the boundary conditions of job insecurity [14]. Scholars may need to modify job insecurity theories to further clarify the distinction between job feature insecurity and job loss insecurity. We believe that theoretical advancements that clarify the unique aspects of job loss insecurity and job feature insecurity will provide more accurate theoretical guidance regarding the sources, consequences, and boundary conditions of different types of job insecurity.

Third, research on workplace gossip is important because gossip constitutes a big proportion of communication in the workplace [43,75]. However, workplace gossip has been largely ignored and is often viewed as socially undesirable and immoral behavior [76]. Recently, scholars have started to recognize the importance of workplace gossip and called for studies that explore the nature and implications of workplace gossip [77,78]. Responding to these calls, we found that the tendency to gossip in the workplace could serve as an emotion-focused coping style that buffers against the negative impact of job insecurity on workplace friendships. Doing so allows us to capture the organizational context that provides the content and triggers of gossip with the ultimate goal to reap the potential benefits of workplace gossip while controlling its negative consequences [41].

Finally, our study contributes to the workplace friendships literature by identifying job insecurity as an antecedent of workplace friendships, as well as the boundary conditions of the job insecurity—workplace friendship relationships. Workplace friendships are unique interpersonal relationships that may serve as an important source of support and intrinsic rewards for employees [79]. Although previous research has predominantly focused on the positive [80,81] and negative outcomes [82] of workplace friendships, it is important to identify the sources of workplace friendships in order to have a deeper understanding of when workplace friendships might emerge.

### 4.2. Practical Implications

Our study has several practical implications. First, our study supports that job insecurity representing a social rejection might negatively impact employees’ workplace friendships, a factor associated with a variety of positive outcomes [80,81]. In order to reduce the negative impact of job insecurity on workplace friendships, organizations could reduce employee job insecurity by increasing the predictability and controllability of their employment. Although job insecurity can be determined by economical forces that are out of employers’ control, organizations can promote open and explicit communication regarding organizational change [83] to increase employees’ perceptions of control and predictability. Organizations may also encourage employees’ participation in decision-making processes and ensure fair procedures are in place at all times in order to help employees to maintain a sense of security [84]. Additionally, organizations may provide attributional training programs that promote employees’ positive attributions for work events (e.g., the change of job features and layoff) so that employees may not attribute their job insecurity to the employer [85].

Although attempts to improve interpersonal relationships in the workplace are less easily controlled by the organization, managers may consider interventions or training programs to promote employees’ conflict management skills to reduce interpersonal conflict [86] in the organization undergoing change (e.g., downsizing).

Finally, workplace gossip is not always bad, as we find gossip can buffer against the negative impact of job insecurity on workplace friendships. Although the tendency to engage in gossip may help employees to cope with job insecurity, gossip (especially negative gossip) is not without negative consequences, such as creating a hostile work environment [87]. Thus, organizations may consider other coping strategies that can help employees to reduce the negative impact of job insecurity on employees’ interpersonal relationships. For instance, organizations may teach employees to use cognitive restructuring strategies to cope with job insecurity via employee training programs [88].

### 4.3. Limitations and Future Research Directions

Despite its theoretical and practical contributions, this study has several limitations with the potential of generating future research. First, this research is a preliminary attempt to understand the interpersonal consequences of both job feature insecurity and job loss insecurity. The present study focused on workplace friendships as the criterion variable. Future research might consider other interpersonal variables in the workplace as outcomes of job insecurity. Moreover, different boundary conditions might exist for the relationships between these two forms of job insecurity and other interpersonal relationships both within and outside work (e.g., relationships with colleagues, family relationships). This is partly supported by our finding that the impact of job feature insecurity and job loss insecurity on workplace friendships might be altered by the tendency to engage in positive gossip and the tendency to engage in negative gossip, respectively.

Second, drawing on the social rejection perspective [19], we propose the main mechanism linking job insecurity and workplace friendships is an intrapersonal withdrawal response. However, because we do not directly examine this social withdrawal responses, we could not rule out the alternative explanations for the relationship between job insecurity and workplace friendships. It is possible that an interpersonal mechanism might also lead to decreased workplace friendships. That is, people who experience job insecurity are less optimal in their social function, which then results in a detrimental effect on their workplace friendships. To gain a better understanding of the underlying explanations for the relationship between job insecurity and workplace friendships, future studies should consider the mediating role of both the interpersonal and intrapersonal processes that might account for the relation between job insecurity on workplace friendships.

Third, we used a single questionnaire to collect all variables of our study from the same source, at the same time. Therefore, common method bias may exist. However, Evans [89] has suggested that common method variance can only alleviate true interactions rather than augmenting artificial interactions. Therefore, our finding of a significant interaction between job feature insecurity and one’s tendency to positive gossip, and a significant interaction between job loss insecurity and one’s tendency to negative gossip, is not likely to be the result of common method bias. Additionally, we collected a convenience sample from Mturk. Although this sample is diverse and the results based on this sample seem to be reliable, our sample might suffer from selection bias—an issue that is common in all convenience samples. We also caution that the results from the unweighted, convenience sample may not represent the population of interest—employees in the United States. As such, the results may have limited external validity and cannot be generalized to the general population. Although we were able to demonstrate our results were robust to employee gender, age, employment status (full- vs. part-time; permanent vs. temporary), and the number of working hours, we were unable to control other, potentially relevant variables (e.g., employee wage and work histories). Similarly, employee personality traits (e.g., extraversion; agreeableness) were not examined nor controlled. Thus, the study may suffer from omitted-variable bias.

Finally, our arguments regarding the directionality of the relationship between job insecurity and workplace friendships are driven by theoretical propositions. However, the cross-sectional design of this study precludes any conclusion regarding the direction of causality. It is possible for those who experience a low-quality workplace relationship to feel insecure about their job. Furthermore, the cross-sectional design also prevents us from gaining a holistic understanding of the trajectory of the relationship between job insecurity and workplace friendships across time. To provide a more comprehensive picture of the relationship between job insecurity and workplace friendships, future research might adopt a longitudinal design to observe how the relationships between job insecurity and workplace friendships may vary over time.

## 5. Conclusions

In the 21st century, job insecurity is prevalent and has been associated with a number of negative personal and organizational outcomes [8]. Extending this line of research, we focused on an interpersonal consequence of job insecurity—workplace friendships. Our findings indicate that when individuals experience job insecurity in the form of job feature insecurity and/or job loss insecurity, they tend to withdraw in their interaction with organizational members and experience a decrease in workplace friendships. We further emphasize that the tendency to positive and negative gossip is likely to buffer against the negative effect of job feature insecurity and job loss insecurity on workplace friendships, respectively. The present research paves the way for future theoretical and empirical endeavors that seek to understand the interpersonal implications of the two forms of job insecurity, and how this negative impact might be buffered by different coping strategies.

## Figures and Tables

**Table 1:** Descriptive statistics and interscale correlations of study variables.

Variable	*M*	*SD*	1	2	3	4	5	6	7	8	9
1. Job Loss Insecurity	0.69	1.05	---								
2. Job Feature Insecurity	3.05	1.26	0.67 **	---							
3. Tendency to Negative Gossip	2.07	0.81	0.08	0.19 **	---						
4. Tendency to Positive Gossip	3.38	0.85	−0.14 *	−0.28 **	0.10	---					
5. Workplace Friendships	5.01	1.48	−0.32 **	−0.52 **	−0.03	0.33 **	---				
6. Gender	1.52	0.50	0.03	0.07	0.01	0.15 *	0.02				
7. Age	37.77	10.56	−0.08	−0.09	−0.10	0.13 *	0.00	0.05			
8. Full- vs. part-employment	1.06	0.25	0.11	0.07	0.03	−0.06	−0.04	0.13 *	−0.08		
9. Permanent vs. Temporary	1.01	0.11	0.13 *	0.14 *	0.02	−0.13 *	−0.16 **	0.03	−0.05	−0.03	
10. # of Hours	40.14	6.77	−0.12	−0.08	0.03	0.07	0.05	−0.12	0.10	−0.64 **	−0.03

Note. * *p* < 0.05. ** *p* < 0.01. M= Mean; SD= Standard deviation; Gender: 1 = Male; 2 = Female; Full- (1) vs. part-employment (2); Permanent (1) vs. Temporary (2).

**Table 2:** Regression analysis results.

Step and Variable	Workplace Friendships	
Step 1	(*df*s = 3, 261)	(*df*s = 3, 261)
Job Loss Insecurity	−0.28 ***	
Job Feature Insecurity		−0.47 ***
Tendency to Positive Gossip	0.29 ***	0.19 ***
Tendency to Negative Gossip	−0.04	0.05
*R* ^2^	0.18	0.31
*F*	19.64	38.25
*p*	0.000	0.000
Step 2	(*df*s = 5, 259)	(*df*s = 5, 259)
Job Loss Insecurity	−0.27 ***	−0.42 ***
Job Feature Insecurity		
Tendency to Positive Gossip	0.31 ***	0.12 ^+^
Tendency to Negative Gossip	−0.04	0.04
Job Loss Insecurity X Positive Gossip	0.09	
Job Loss Insecurity X Negative Gossip	0.10 ^+^	
Job Feature Insecurity X Positive Gossip		0.16 *
Job Feature Insecurity X Negative Gossip		0.01
Δ *R*^2^	0.02	0.01
Δ *F*	3.50	2.71
*p*	0.032	0.069
Step 3	(*df*s = 10, 254)	(*df*s = 10, 254)
Job Loss Insecurity	−0.26 ***	−0.43 ***
Job Feature Insecurity		
Tendency to Positive Gossip	0.31 ***	0.12 ^+^
Tendency to Negative Gossip	−0.05	0.03
Job Loss Insecurity X Positive Gossip	0.08	
Job Loss Insecurity X Negative Gossip	0.12 *	
Job Feature Insecurity X Positive Gossip		0.15 *
Job Feature Insecurity X Negative Gossip		0.01
Gender (1 = Male; 2 = Female)	−0.03	0.02
Age	−0.07	−0.07
Full- (1) vs. part-employment (2)	0.00	−0.00
Permanent (1) vs. Temporary (2)	−0.06	−0.05
# of hours	0.10	0.02
Δ *R*^2^	0.01	0.01
Δ *F*	0.58	0.52
*p*	0.72	0.76

*Notes*: ^+^*p* <0.10. * *p* < 0.05, ** *p* < 0.01, *** *p* <0.001.
